# Photodiode-based time zero determination for ultrafast electron microscopy

**DOI:** 10.1063/4.0000218

**Published:** 2023-11-06

**Authors:** S. T. Kempers, S. Borrelli, E. R. Kieft, H. A. van Doorn, P. H. A. Mutsaers, O. J. Luiten

**Affiliations:** 1 Eindhoven University of Technology, Coherence and Quantum Technology, 5600 MB Eindhoven, the Netherlands; 2 Thermo Fisher Scientific, Achtseweg Noord 5, 5651 GC Eindhoven, The Netherlands

## Abstract

Pump-probe experiments in ultrafast electron microscopy require temporal overlap between the pump and probe pulses. Accurate measurements of the time delay between them allows for the determination of the time zero, the moment in time where both pulses perfectly overlap. In this work, we present the use of a photodiode-based alignment method for these time zero measurements. The cheap and easy-to-use device consists of a photodiode in a sample holder and enables us to temporally align individual, single-electron pulses with femtosecond laser pulses. In a first device, a temporal resolution of 24 ps is obtained, limited by the photodiode design. Future work will utilize a smaller photodiode with a lower capacitance, which will increase the temporal resolution and add spatial resolution as well. This upgrade will bring the method toward the micrometer and picosecond spatiotemporal resolution.

## I. INTRODUCTION

Ever since the realization of the first ultrafast transmission electron microscope (UTEM),^1^ interest in capturing ultrafast dynamics has risen immensely.^2^ This led to the development of completely new measurements techniques, such as D-TEM,^3^ PINEM,^4^ movie-mode TEM,^5^ PEEM,^6^ and EEGS.^7^ These experimental methods all rely on a trigger exciting the dynamics to be investigated in a sample. Typically, femtosecond oscillators are used to generate the trigger pulses (pump). The dynamics are then captured by the electron pulse (probe) after a certain time delay. These pump-probe experiments allow for the investigation of samples on the sub-picosecond timescale^8,9^ and even enable the creation of real-time movies of ultrafast dynamics, such as phonons.^10^ UTEMs have two modes of operation, such as stroboscopic mode and single-shot mode. In the stroboscopic mode, reversible dynamics are observed by repeated measurements of low dose electrons pulses, ranging from less than one electron up to a thousand electrons per pulse. The stroboscopic approach has been used to reveal order–disorder transitions in molecules,^11^ visualize surface plasmon polaritions,^12^ capture plasmons at buried interfaces,^13^ image 2D polariton dynamics,^14^ investigate phase transistions in Mott insulators,^15^ and mapping of the order parameter during a phase transition.^16^ Contrary to this is the single-shot mode, where the number of electrons per pulse is much higher ( $>10^{7}$), such that only a single electron exposure is necessary, enabling the investigation of irreversible ultrafast dynamics. An important aspect of realizing ultrafast pump-probe experiments is to have an accurate determination of time zero, i.e., the exact moment in time where the two types of pulses temporally overlap. This time zero is usually found using laser-induced plasmas,^17^ ponderomotive scattering,^18^ or laser-triggered blankers.^19^ These processes typically have some inherent form of delay between the impact of the incident pulse and the signal generated and require temporal overlap between the pulses to obtain a signal. We, therefore, developed a method that allows for a direct measurement of the time delay between laser pulses and single electron pulses, without relying on any interaction between the two. At the moment a resolution of 24 ps is achieved, but improvements are possible. The method utilizes a photodiode attached to a standard transmission electron microscope (TEM) sample holder, allowing it to be placed directly inside the beam path. The generation of charge induced by the photons and the individual electrons is measured by an oscilloscope, allowing the capture of real-time signals. This creates an easy to use device, which allows for single-shot time zero measurements.

## II. METHODS

### A. Cavity-based electron microscope

**FIG. 1.**
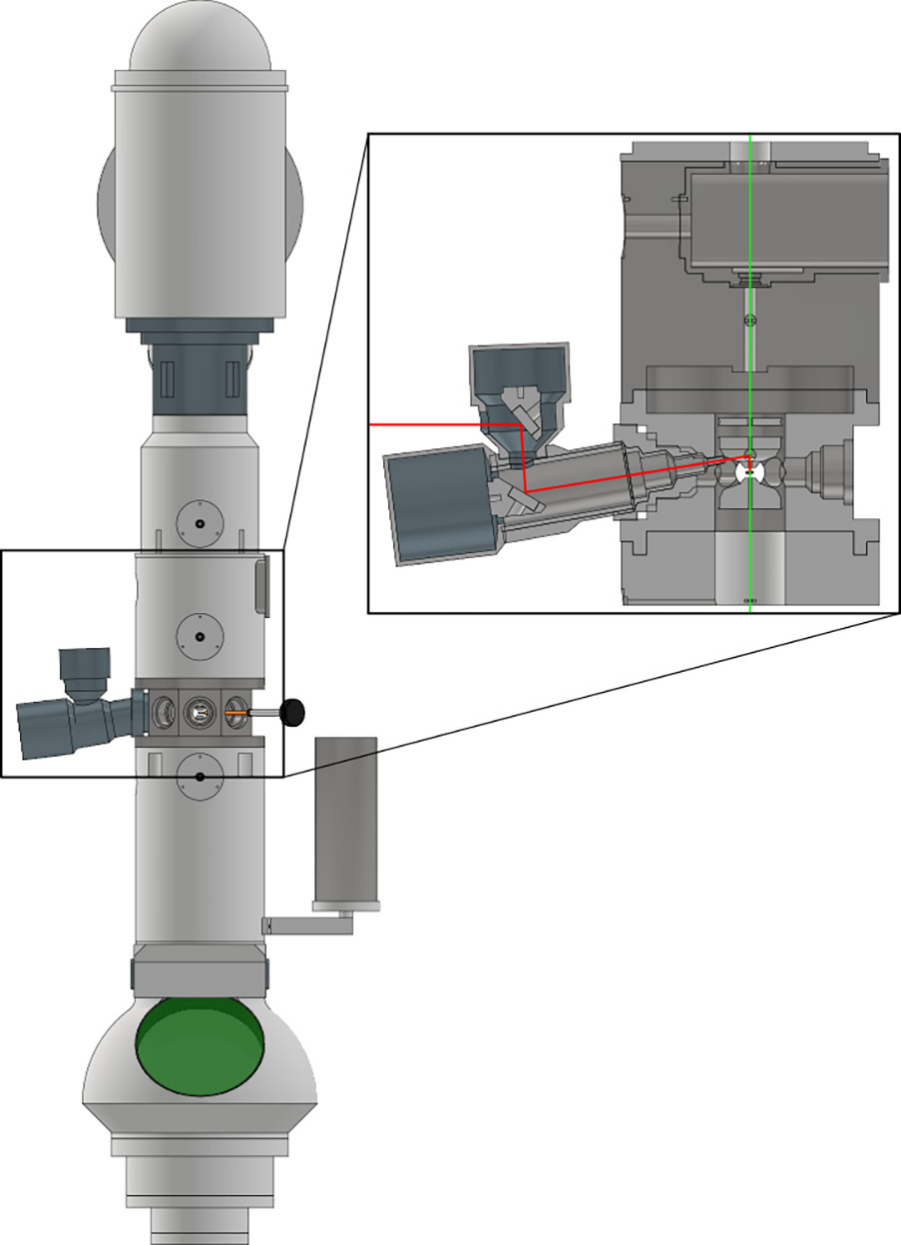
Schematic overview of the electron microscope with the laser insertion module on the left. The inset shows a close-up of the sample plane, with the laser (red) entering the microscope from the left, and the electrons (green) coming in from the top. The darker gray area above the sample chamber is the extension of the microscope and houses the microwave cavity. Drawing is illustrative and not to scale.

**FIG. 2.**
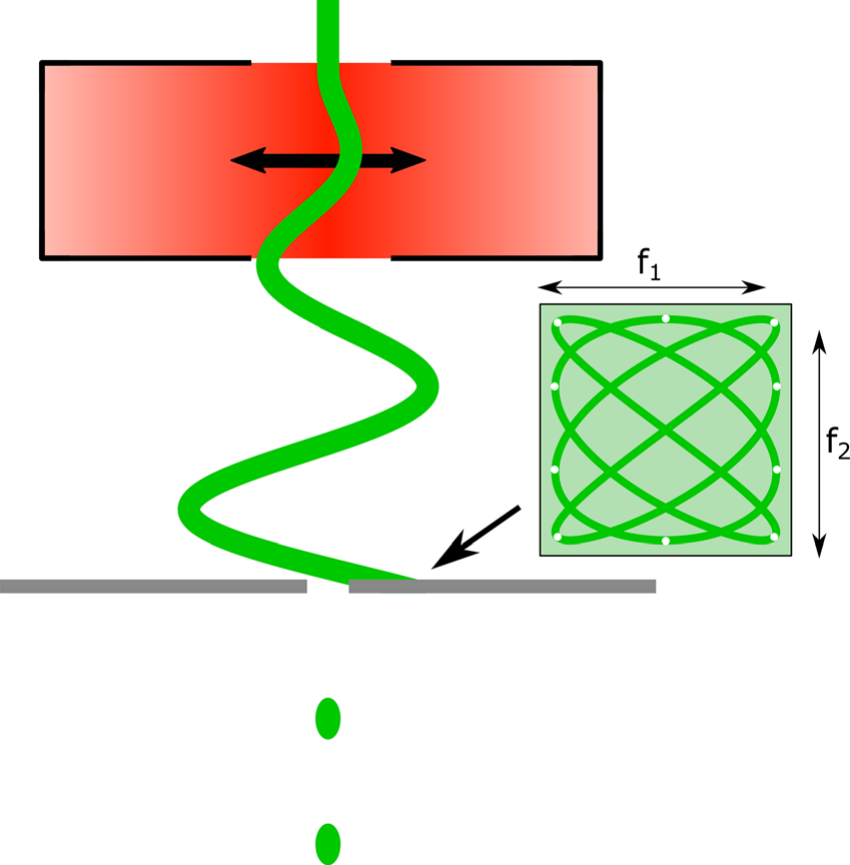
Visualization of 3 GHz streaking of the electron beam by a TM_110_ microwave cavity. The inset shows the Lissajous pattern traced by the cavity when operated in dual-mode configuration.

The first part of the setup consists of a cavity-based UTEM, as described by Verhoeven *et al.*^20,21^ and Borrelli *et al.*^22^ The cavity-based UTEM is a modified 200 keV Thermo Fisher Tecnai F20, a schematic of which can be seen in Fig. 1, in which the column is extended by 20 cm. The extension houses a 3 GHz pillbox cavity operating in the TM_110_ mode. The oscillating magnetic field of this mode is used to streak the continuous electron beam across an aperture, creating single-electron pulses with pulse lengths down to 100 fs (as illustrated in Fig. 2). During the measurements, the microscope is set to produce 1 ps electron pulses at a repetition rate of 75 MHz, with an average current of 27 fA, leading to less than one electron per pulse on average. While a relatively low current is used in these experiments, the photodiode-based alignment method is not limited by the current, so higher values can be used as well.

The second part of the setup is a femtosecond Ti:sapph oscillator, which produces 20 fs laser pulses at a repetition rate of 75 MHz with an average power of 400 mW. The optical path enters the microscope at the sample plane and enters in such a way that the laser pulses and electron pulses travel under a small angle with respect to each other ( ∼2°). A 30 cm optical delay line is used to shift the arrival time of the laser pulses over a 2 ns range.

In order to match the electron pulse repetition rate to the 75 MHz repetition rate of the laser, a dual-mode deflection cavity is used. This cavity uses two orthogonal TM_110_ modes, one oscillating at 3.0 GHz and one at 3.075 GHz. Instead of a sinusoidal streak, the transverse deflection will now trace a Lissajous pattern, which repeats at a frequency of 75 MHz, i.e., 13.3 ns (see Fig. 2). The driving signals for the two modes are derived from the same Ti:Sapph oscillator signal and can, therefore, be accurately phase-locked to each other. The resulting electron pulses are created at the different frequency of the two modes, which matches the laser frequency, and are synchronized to within 100 fs to the laser pulses. Further details on the dual-mode cavity can be found in Van Rens *et al.*^23^ The two driving microwave signals can be delayed in time with respect to one another by using two phase shifters. This allows the electron pulse to be shifted in time over the whole 13.3 ns period of the Lissajous pattern, which would otherwise require a four meter long optical delay line.

Contrary to photoemission-based UTEMs, in a cavity-based UTEM, the electron pulses are not automatically synchronized to the laser pulses, as they are not directly created by the laser. This makes the photodiode-based alignment method especially suited for the cavity-based UTEM, since the single-shot method allows for time difference determination even without an external synchronization system. The high dynamic range allows both types of pulses to be found in a large time window (13.3 ns), while maintaining the sub-ns resolution.

### B. Photodiode-based temporal alignment

**FIG. 3.**
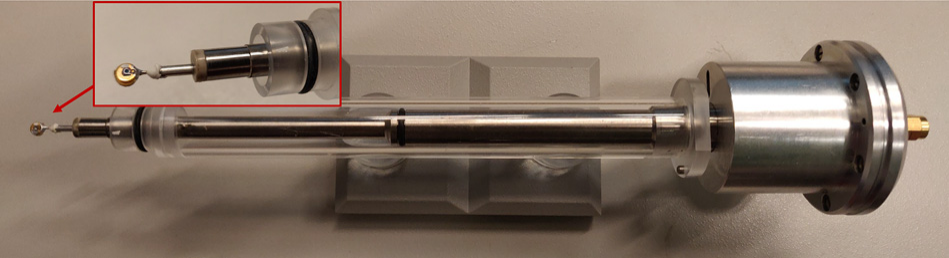
Modified sample holder with a photodiode attached to the end of the holder, as shown in the inset.

The photodiode-based alignment device is based on a modified TEM single tilt sample holder, an image of which can be seen in Fig. 3. A Centronic AEPX65 photodiode with 6 pF capacitance and an active area of 0.55 mm^2^ is attached to the tip of the sample holder. A coax cable connects the photodiode to an SMA connection on the other end of the sample holder (outside vacuum). An O-ring preserves the vacuum between the outside end of the coax and the end attached to the photodiode. The outside end (outside the microscope) of the sample holder is adjustable to remove tension on the coax cable, which arises from connecting electronic components to the sample holder. A Mini-Circuits ZX85-12G-S+ bias-tee (bandwidth from 0.2 to 12 000 MHz) is used to apply a 5 V reverse bias to the photodiode. The output signal of the photodiode is amplified (20 dB) by a Mini-Circuits ZX60-33LN-S+ amplifier (bandwidth from 50 to 3000 MHz). The signal is then sent to an Agilent Infinium 54845A oscilloscope (8 GSa/s and 1.5 GHz bandwidth). The oscilloscope is triggered by the photodiode signal. Part of the laser signal used for the synchronization of the electron pulses is used as a clock reference on the oscilloscope.

## III. RESULTS

### A. Arrival time

**FIG. 4.**
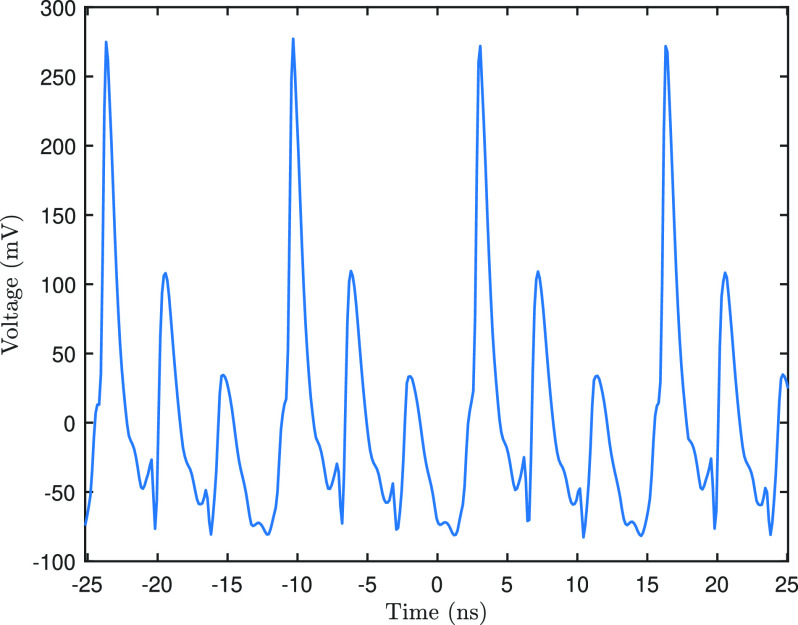
The voltage signal of four laser pulses impinging on the photodiode. The highest ( ∼250 mV) peaks, which repeat every 13.3 ns, indicate the arrival of the laser pulses. The subsequent lower peaks are caused by reflections inside the sample holder.

Figure 4 shows an example of an obtained signal when illuminating the photodiode with the laser pulses. The signal repeats itself every 13.3 ns. The highest voltage peak indicates the arrival of photons on the detector, while the subsequent lower peaks show the effect of reflections in the coax cable inside the sample holder. Determining the exact arrival time of the laser pulse (relative to the clock signal) requires knowledge on the photon to electron conversion processes that occur inside the photodiode. One would also have to include the delay of all electronic components and cables between the source of the signal and the oscilloscope. Fortunately, with pump-probe experiments, one is usually interested only in the relative time delay between the electrons and the laser pulse. The advantage of using this photodiode method is that the electrons and the laser pulse are measured at the same location, with the same electronic components. Comparing the individual signals to a clock signal, therefore, allows us to determine the time zero.

**FIG. 5.**
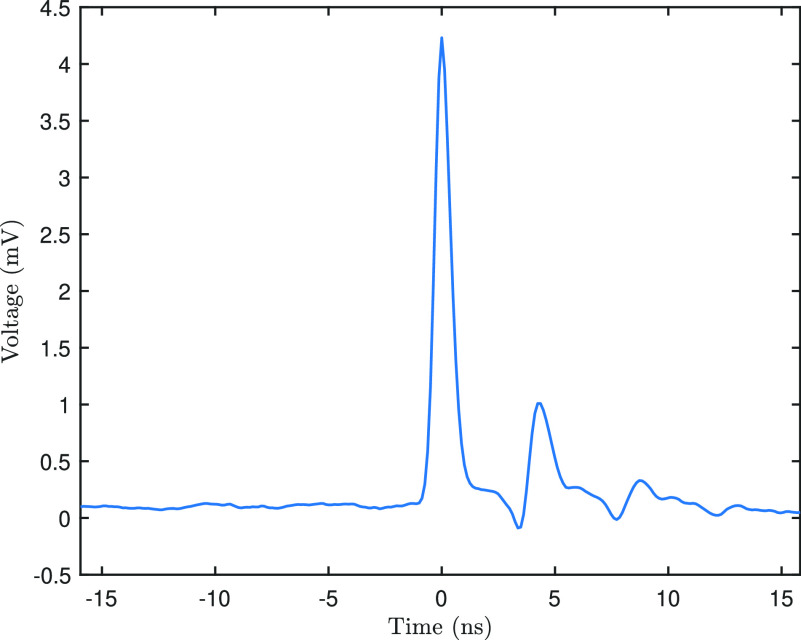
The voltage signal of an electron pulse striking the photodiode. Similar to the laser signal, the ∼4 mV peak indicates the electron arrival, while the lower peak roughly 4 ns later is due to reflections in the sample holder.

A similar analysis can be performed for the electron signal, an example of which is shown in Fig. 5. When the single-electron pulse reaches the photodiode it will induce, on average, a voltage of 4.2 ± 0.7 mV. The width of the generated voltage pulse is 760 ± 94 ps (FWHM). Using the NIST database for the stopping power of Silicon gives an estimate for the voltage generated by a 200 keV electron.^24^ This estimate depends, of course, on the thickness of the active layer of the photodiode. In the case of a 1 *μ*m thick active layer, each electron will induce roughly 80 aC of charge. This translates to a voltage of roughly 6 mV, which matches well with the observed values.

The used current of 27 fA translates into roughly $1.7\times 10^{5}$ electrons per second. This means that with a repetition rate of 75 MHz, there will on average be 1 electron per 500 pulses. Since the photodiode is able to capture these pulses, this indicates that the method is able to detect single-electron pulses.

The arrival times of the electron pulses and the laser pulses are determined relative to the clock signal. The steepest point of the leading edge of the voltage pulse is chosen as the reference point of the arrival time, as this point has the highest sensitivity (i.e., maximum $\frac{\Delta V}{\Delta t}$) and, thus, lowest time uncertainty. Adjusting the arrival time of the electrons using the phases of the cavity, as explained in Sec. II, allows the difference in arrival time of the electrons and the laser pulse to be set within the scannable range of the optical delay line, which is used for the fine tuning.

### B. Timing accuracy

The resolution with which the arrival time of a single voltage pulse can be determined, depends on the maximum slope of the signal. As such, the rise time of the signal is an important figure of merit.^25,26^ The significant contributor to the rise time is the 1.5 GHz bandwidth of the oscilloscope. This bandwidth, *BW*, can be translated to a rise time using the following equation:

$$t_{r}=\frac{0.35}{BW}.$$

Inserting the bandwidth of the scope results in a rise time of 233 ps. This prediction can be compared to the measured rise time (the time between 10% and 90% of the maximum voltage) of the signal. In the case of the electron signals of Fig. 5, a *t_r_* of 392 ± 61 ps is found.

The discrepancy between the expected rise time (based on the oscilloscope bandwidth) and measured one can be attributed to the copper wire connecting the terminals of the photodiode to the coax cable. This connection will effectively add an inductance in the equivalent circuit of the photodiode.^27^ Simple simulations (using SIMetrix) show that an inductance of roughly 10 nH would lead to the observed rise time. This would indicate a 5 mm length of the copper wire (one for ground and one for the potential), assuming a typical 1 nH per mm cable. Using a photodiode with a smaller capacitance will increase the bandwidth of the system, thus reducing the rise time. At that point, the rise time will be limited by the bandwidth of the oscilloscope. Photodiodes with smaller capacitances typically have smaller active areas as well. This will add a spatial resolution to the device as well, making it an even more versatile tool.

The timing resolution of the device due to jitter, *σ_t_*, is deduced from the noise level *σ*_noise_ and the maximum voltage slope ${\left(\frac{dV}{dt}\right)}_{\text{max}}$ as follows:^26,27^

$$\sigma_{t}=\frac{\sigma_{\text{noise}}}{{\left(\frac{dV}{dt}\right)}_{\text{max}}}\approx \frac{\sigma_{\text{noise}}t_{r}}{0.8V_{\text{max}}},$$

which can also be related to the rise time of the signal via $\frac{dV}{dt}\approx \frac{0.8V_{\text{max}}}{t_{r}}$, with *V*_max_ being the maximum value of the signal. Using a root mean square noise of 0.2 mV (as deduced form the baseline in Fig. 5), we obtain a temporal resolution of 24 ps.

**FIG. 6.**
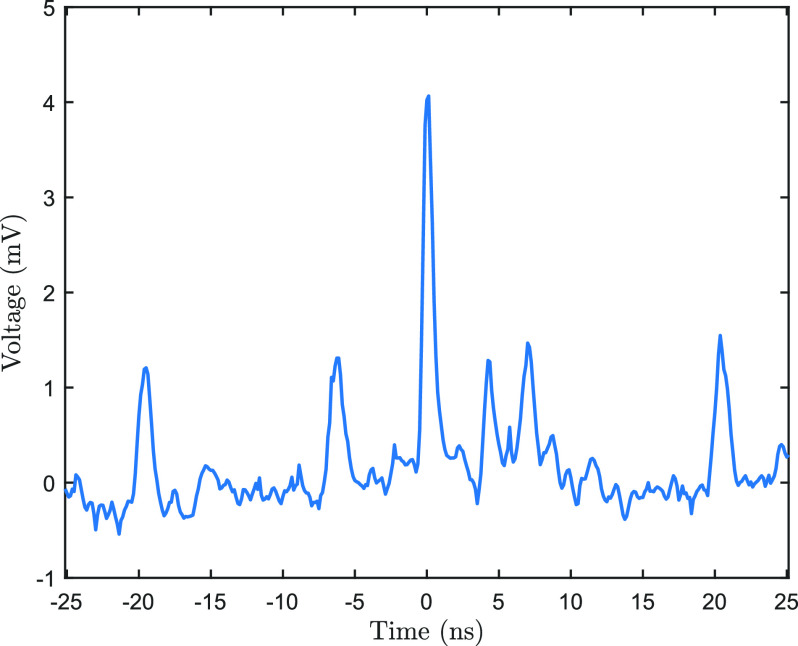
Example signal of both laser and electron pulses hitting the photodiode. The laser is attenuated to allow triggering on the single-electron pulse, which is consequently located around t≈0. One reflection of the 4 mV electron signal is visible around *t* = 4 ns, the remaining four peaks are laser pulses.

The device can also be used for single-shot measurements by letting the laser and electron pulses impinge on the photodiode simultaneously. Attenuating the photon signal will reduce the number of reflections visible, while also enabling the oscilloscope to trigger on the electron signal. The obtained waveforms, such as the one in Fig. 6, show both the arrival of the laser pulse (the 1 mV peaks) as well as the electron pulse (the 4 mV peak at t≈0). This, then, allows for direct observation of the time delay between the pulses, at the interaction point.

## IV. CONCLUSIONS AND OUTLOOK

With the use of the photodiode on a modified sample holder, a determination of the time zero within 24 ps is possible. This single-shot method allows for direct detection of photons and single electrons by the same device at the same location and time. This eliminates the uncertainty arising from correlating some electron signal to a different photon signal. By incorporating the photodiode design into a standard sample holder, the device becomes plug-and-play and does not require extensive vacuum pumping of the system. Using a photodiode with a smaller active area will reduce the capacitance and will, therefore, lower the rise time of the voltage signal. Off-the-shelf photodiodes can have capacitances up to two orders of magnitude lower, pushing the resolution down to the single picosecond level. The bandwidths of the rest of the system should be matched to this increased resolution. The lower surface area of these photodiodes (diameter < 50 *μ*m) will also add the possibility of using the device as a spatial alignment tool. The photodiode-based method will then reach the regime of micrometer-picosecond spatiotemporal resolution.

For pump-probe measurements only the arrival time of a signal is of importance, so there is no need to capture the complete temporal profile, such as the ones shown in Figs. 4 and 5. The oscilloscope is, therefore, an unnecessary bandwidth limitation. Future implementations of the method might be better off utilizing a different data acquisition device. Using dedicated high speed timing electronics, such as, for example, nuclear instrumentation modules (NIMs), enables obtaining timing information without the use of an oscilloscope. Other approaches of determining the time zero could be to use the non-linearity of the detector to detect the overlap of the two pulses or to use the high bandwidth of a spectrum analyzer to detect changes in the spectrum of the output signal. The ease-of-use and the straightforward data interpretation of the device allow for quick and accurate time zero determination for pump-probe experiments in electron microscopy.

## Data Availability

The data that support the findings of this study are available from the corresponding author upon reasonable request.
